# Supplementary material for: Spinal Excitatory Dynorphinergic Interneurons Contribute to Burn Injury-Induced Nociception Mediated by Phosphorylated Histone 3 at Serine 10 in Rodents
Source: Int J Mol Sci. 2021 Feb 25;22(5):2297. doi: 10.3390/ijms22052297 (PMC7956488; doi:10.3390/ijms22052297)
Supplement: Supplementary file 1 [file ijms-22-02297-s001.pdf]

Spinal Excitatory Dynorphinergic Interneurons Contribute to Burn Injury-Induced Nociception Mediated by Phosphorylated Histone 3 at Serine 10 in Rodents

Angelika Varga [1,2,*], Zoltán Mészár [2], Miklós Sivadó [1,2], Tímea Bácskai [3], Bence Végh [2], Éva Kókai [1,2], István Nagy [4,5] and Péter Szücs [1,2]

[1] MTA-DE-NAP B-Pain Control Research Group, University of Debrecen, 4032 Debrecen, Hungary; miklos.sivado@anat.med.unideb.hu (M.S.); kokai.eva@med.unideb.hu (E.K.); szucs.peter@med.unideb.hu (P.S.)

[2] Department of Anatomy, Histology and Embryology, Faculty of Medicine, University of Debrecen, 4032 Debrecen, Hungary; meszarz@anat.med.unideb.hu (Z.M.); vegbence1997@gmail.com (B.V.)

[3] Division of Dental Anatomy, Department of Basic Medical Sciences, Faculty of Dentistry, University of Debrecen, 4032 Debrecen, Hungary; bacskai.timea@anat.med.unideb.hu

[4] Department of Surgery and Cancer, Imperial College London, London SW7 ZAZ, UK; i.nagy@imperial.ac.uk

[5] Department of Physiology, Faculty of Medicine, University of Debrecen, 4032 Debrecen, Hungary

* Correspondence: varga.angelika@med.unideb.hu; Tel.: +36-(52)-255-567

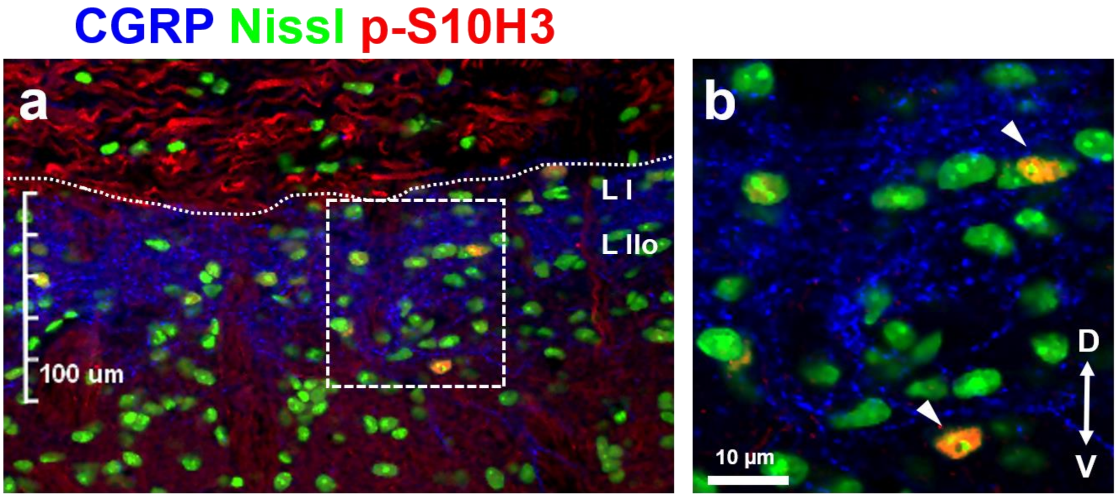

Figure S1. The termination zone of CGRP-containing peptidergic afferents outlines the area where colocalization analyses was carried out. (**a**) Immunostaining with antibodies against p-S10H3 (red), Nissl (green) and CGRP (blue) in a single plane from a parasagittal section of spinal cord from a wild-type mouse. Colocalization analyses were performed till a depth of 100 um in the superficial dorsal horn where peptidergic CGRP fibers arborize (LI & LIIo). Vertical scale bar indicates the total width of CGRP band. Dotted line indicates the border between the gray and white matter. (**b**) At higher magnification, arrowheads indicate p-S10H3-positive neurons which are surrounded by CGRP immunoreactive afferents. D, dorsal; V, ventral.

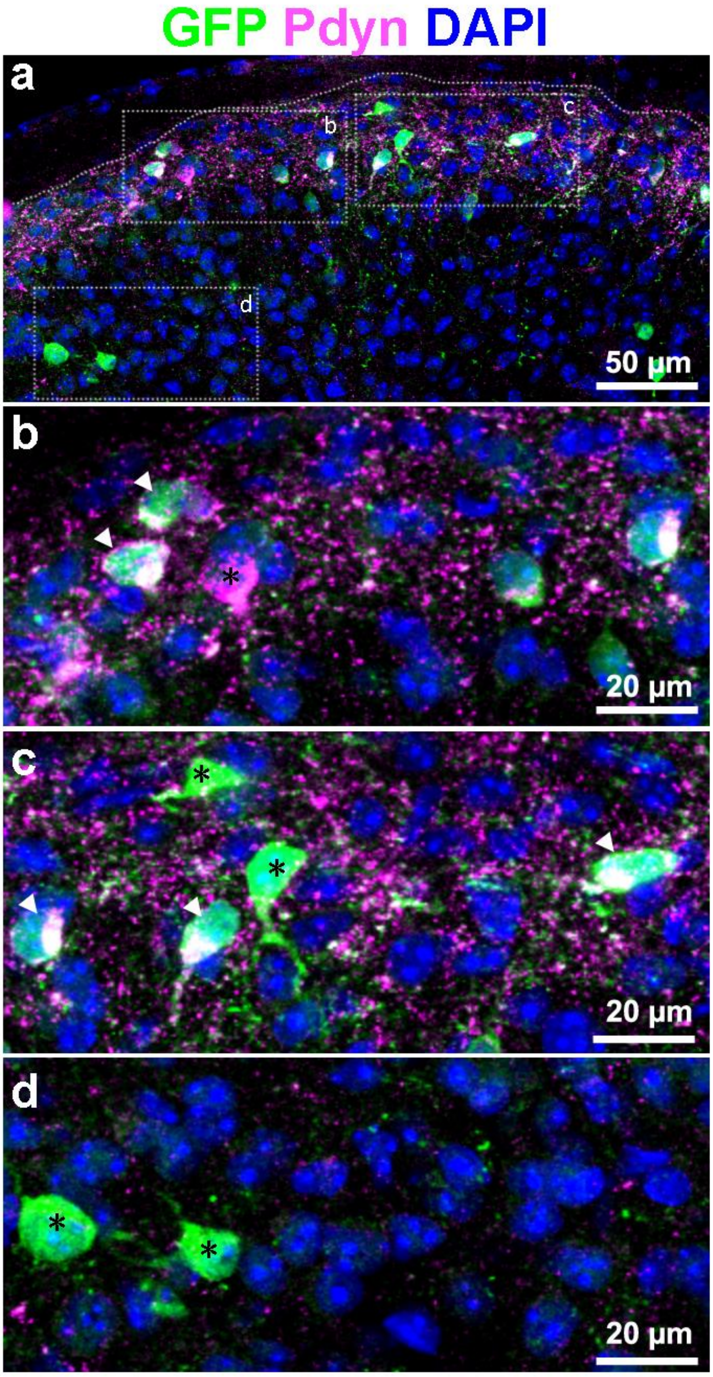

Figure S2. Validation of the Pdyn:EGFP hybrid strain for neurochemical characterization of dynorphinergic (Pdyn) neurons in the superficial dorsal horn of spinal cord. (**a**) A representative image showing immunostaining for GFP (green), Pdyn (magenta) and cell-nuclei specific DAPI (blue) in a transverse spinal cord section of Pdyn:EGFP mouse. In this hybrid mouse Pdyn expression is linked to cas9-EGFP due to cre-dependence of cas9. Hence, virtually all dynorphinergic neurons should exhibit GFP-IR in their cytoplasm. (**b**–**d**) In the insets, most superficial laminae the majority of EGFP+ cells were Pdyn-IR neurons (arrowheads; b,c). The occasional EGFP+ neurons in laminae I and IIo that lacked Pdyn-IR, were very few in number (asterisks; b-c). EGFP+/Pdyn- neurons were more numerous in deeper dorsal horn laminae (d) indicating that these neurons probably transiently expressed Pdyn at any earlier stage of their development.

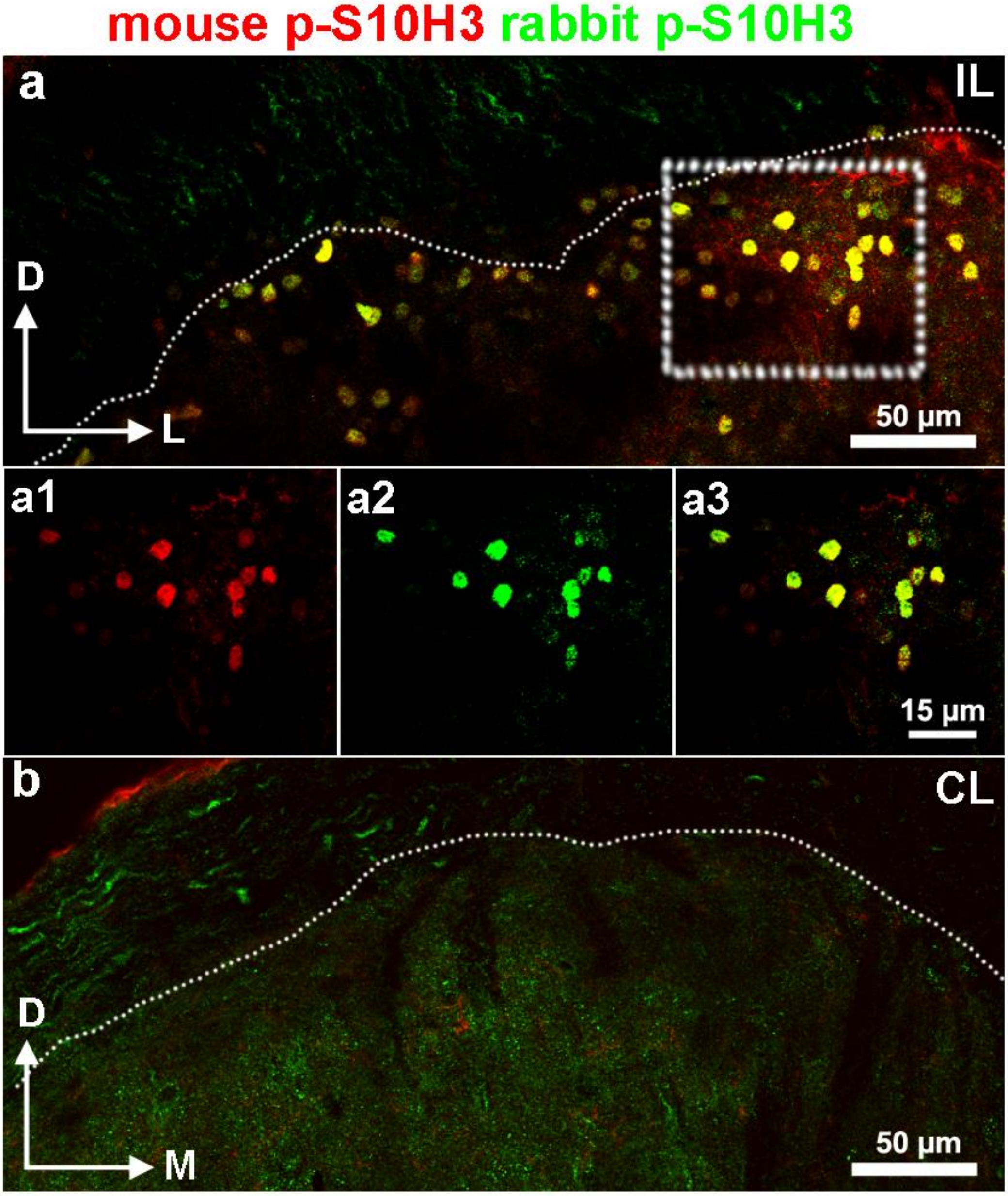

Figure S3. Verification of the specificity of anti-p-S10H3 antibodies used in the study. A representative images showing immunostaining for the anti-p-S10H3 antibody produced in mouse (red) and in rabbit (green) in a transverse spinal cord section of a wild-type mouse, representing the ipsilateral (IL; **a**) and contralateral (CL; **b**) side of the burn injury. Insets (**a1**–**a3**) show higher magnification view of the area designated by a square on image (a). The two antibodies against p-S10H3 exhibit close to identical colocalization pattern on the IL side of burn injury. The contralateral side showed a complete lack of immunolabeling. Dotted line indicates the border between the gray and white matter. D, dorsal; M, medial; L, lateral.

Table S1. Distribition of calretinin+ and dynorphin+ neurons showing p-S10H3 in their nuclei, among excitatory and inhibitory SDH neuronal pools, identified by RFP immunoreactivity in VGAT- or Vglut2:tdTomato transgenic mice.

	**% that were CR+**	**% that were Pdyn+**
VGAT	36.0%<br>(n = 10)	20.4%<br>(n = 6)
Vglut2	75.0%<br>(n = 7)	47.2%<br>(n = 7)

Number of sections is shown in brackets from VGAT:tdTomato or Vglut2:tdTomato transgenic mice (n=2 and 3, respectively).
CR+ and Pdyn+ mean calretinin and prodynorphin-IR neurons, respectively.
Percentages were calculated as follows: total number of triple labelled neurons (CR+ or Pdyn+ / p-S10H3+ / VGAT+ or Vglut2+) was divided by the total number of CR+ or Pdyn+ neurons showing p-S10H3-IR.
